# *catena*-Poly[[dimethyl­tin(IV)]-μ-*cis*-cyclo­hexane-1,2-dicarboxyl­ato]

**DOI:** 10.1107/S1600536809004097

**Published:** 2009-02-11

**Authors:** Yuerong Wang, Rufen Zhang, Yongxin Li

**Affiliations:** a Department of Chemistry, Liaocheng University, Liaocheng 252059, People’s Republic of China

## Abstract

The title complex, [Sn(CH_3_)_2_(C_8_H_10_O_4_)]_*n*_, was synthesized from *cis*-cyclo­hexane-1,2-dicarboxylic acid and dimethyl­tin dichloride. The complex has a bridging bis-bidentate carboxyl­ate group resulting in a zig-zag chain structure parallel to [001]. The Sn atom is six-coordinated and displays a distorted octa­hedral geometry.

## Related literature

For background to organotin complexes, see: Gielen (2002 ▶); Han *et al.* (2007 ▶). For related structures, see: Swisher *et al.* (1984 ▶).
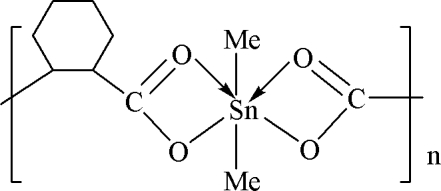


## Experimental

### 

#### Crystal data

[Sn(CH_3_)_2_(C_8_H_10_O_4_)]
*M* *_r_* = 318.92
Monoclinic, 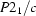

*a* = 10.0880 (16) Å
*b* = 10.430 (2) Å
*c* = 11.592 (2) Å
β = 99.041 (2)°
*V* = 1204.5 (4) Å^3^
*Z* = 4
Mo *K*α radiation
μ = 2.11 mm^−1^
*T* = 298 (2) K
0.32 × 0.19 × 0.17 mm

#### Data collection

Bruker SMART CCD area-detector diffractometer
Absorption correction: multi-scan (*SADABS*; Sheldrick, 1996 ▶) *T*_min_ = 0.551, *T*_max_ = 0.715
6188 measured reflections
2117 independent reflections
1822 reflections with *I* > 2σ(*I*)
*R*_int_ = 0.022

#### Refinement

*R*[*F* ^2^ > 2σ(*F* ^2^)] = 0.031
*wR*(*F* ^2^) = 0.062
*S* = 1.19
2117 reflections
136 parameters
H-atom parameters constrained
Δρ_max_ = 0.52 e Å^−3^
Δρ_min_ = −0.43 e Å^−3^

### 

Data collection: *SMART* (Bruker, 1996 ▶); cell refinement: *SAINT* (Bruker, 1996 ▶); data reduction: *SAINT*; program(s) used to solve structure: *SHELXS97* (Sheldrick, 2008 ▶); program(s) used to refine structure: *SHELXL97* (Sheldrick, 2008 ▶); molecular graphics: *SHELXTL* (Sheldrick, 2008 ▶); software used to prepare material for publication: *SHELXTL*.

## Supplementary Material

Crystal structure: contains datablocks I, global. DOI: 10.1107/S1600536809004097/bx2192sup1.cif

Structure factors: contains datablocks I. DOI: 10.1107/S1600536809004097/bx2192Isup2.hkl

Additional supplementary materials:  crystallographic information; 3D view; checkCIF report

## Figures and Tables

**Table:** 

Sn1—O3	2.089 (3)
Sn1—C9	2.089 (4)
Sn1—C10	2.098 (4)
Sn1—O1	2.102 (3)
Sn1—O4	2.570 (3)
Sn1—O2	2.660 (3)

**Table:** 

C9—Sn1—C10	137.14 (18)

**Fig. 1.**
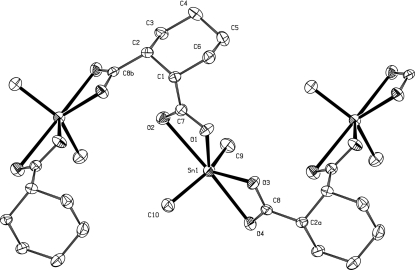
Part of the structure of (I) showing one-dimensional extended polymeric network, with the atom-numbering scheme. Displacement ellipsoids are drawn at the 30% probability level and H atoms have been omitted for clarity. [Symmetry codes: (a) x, 1/2 - y, -1/2 + z; (b) x, 1/2 - x, 1/2 + z]

**Fig. 2.**
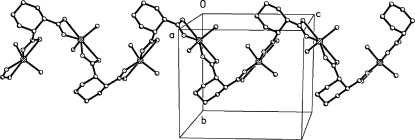
The one-dimensional zigzag chain of the title complex

## supplementary crystallographic information

### Comment

Recently, organotin complexes have been attracting increasing attention partly owing to their determinately or potentially pharmic value, which have been reported many before (Gielen, 2002), and also for the versatile molecular structure and supramolecular architecture exhibited by these complexes (Han *et al.*, 2007). In order to explore the relationships between the properties and structures, we report here the structure of the title complex. Fig. 1 the structure of (I) showing one-dimensional extended polymeric network, and the one-dimensional chain along [001] direction of complex is shown in Fig. 2. Sn atom is six coordinated and displays a octahedral distorted geometry.

### Experimental

The reaction was carried out under nitrogen atmoshpere. *cis*-cyclohexane-1,2-dicarboxylic acid (0.173 g, 1 mmol) was added to the solution of benzene(30 ml) with sodium ethoxide (0.136 g, 2 mmol) in a Schlenk flask. After stirring for 10 min, dimethyltin dichloride (0.220 g, 1 mmol) was added to the mixture. The mixture was kept at 328 K for 12 h. After cooling down to the room temperature, the solution was filtered. The solvent of the filtrate was gradually removed by evaporation under vacuum until a solid product was obtained. The solid was then recrystallized from aether. Colorless single crystals of the title complex were obtained after one week. Yield, 86%. Analysis calculated for C_10_H_16_O_4_Sn: C 48.76, H 6.55; found: C 48.66, H 6.68. The elemental analyses were performed with PERKIN ELMER MODEL 2400 SERIES II.

### Refinement

All H atoms were placed in geometrically idealized positions methyl (C—H = 0.96 Å), methylene (C—H = 0.97 Å), (C—H = 0.98 Å) and treated as riding on their parent atoms, with *U*_iso_(H) = 1.5*U*_eq_(CH_3_), *U*_iso_(H) = 1.2*U*_eq_(CH_2_), *U*_iso_(H) = 1.2*U*_eq_(CH).

### Crystal data

**Table:**

[Sn(CH_3_)_2_(C_8_H_10_O_4_)]	*F*(000) = 632
*M**_r_* = 318.92	*D*_x_ = 1.759 Mg m^−^^3^
Monoclinic, *P*2_1_/*c*	Mo *K*α radiation, λ = 0.71073 Å
Hall symbol: -P 2ybc	Cell parameters from 4238 reflections
*a* = 10.0880 (16) Å	θ = 2.7–28.3°
*b* = 10.430 (2) Å	µ = 2.11 mm^−^^1^
*c* = 11.592 (2) Å	*T* = 298 K
β = 99.041 (2)°	Block, colourless
*V* = 1204.5 (4) Å^3^	0.32 × 0.19 × 0.17 mm
*Z* = 4	

### Data collection

**Table:**

Bruker SMART CCD area-detector diffractometer	2117 independent reflections
Radiation source: fine-focus sealed tube	1822 reflections with *I* > 2σ(*I*)
graphite	*R*_int_ = 0.022
φ and ω scans	θ_max_ = 25.0°, θ_min_ = 2.0°
Absorption correction: multi-scan (*SADABS*; Sheldrick, 1996)	*h* = −11→12
*T*_min_ = 0.551, *T*_max_ = 0.715	*k* = −11→12
6188 measured reflections	*l* = −11→13

### Refinement

**Table:**

Refinement on *F*^2^	Primary atom site location: structure-invariant direct methods
Least-squares matrix: full	Secondary atom site location: difference Fourier map
*R*[*F*^2^ > 2σ(*F*^2^)] = 0.031	Hydrogen site location: inferred from neighbouring sites
*wR*(*F*^2^) = 0.062	H-atom parameters constrained
*S* = 1.19	*w* = 1/[σ^2^(*F*_o_^2^) + (0.0181*P*)^2^ + 1.262*P*] where *P* = (*F*_o_^2^ + 2*F*_c_^2^)/3
2117 reflections	(Δ/σ)_max_ = 0.001
136 parameters	Δρ_max_ = 0.52 e Å^−^^3^
0 restraints	Δρ_min_ = −0.43 e Å^−^^3^

### Special details

**Table:**

Geometry. All e.s.d.'s (except the e.s.d. in the dihedral angle between two l.s. planes) are estimated using the full covariance matrix. The cell e.s.d.'s are taken into account individually in the estimation of e.s.d.'s in distances, angles and torsion angles; correlations between e.s.d.'s in cell parameters are only used when they are defined by crystal symmetry. An approximate (isotropic) treatment of cell e.s.d.'s is used for estimating e.s.d.'s involving l.s. planes.
Refinement. Refinement of *F*^2^ against ALL reflections. The weighted *R*-factor *wR* and goodness of fit *S* are based on *F*^2^, conventional *R*-factors *R* are based on *F*, with *F* set to zero for negative *F*^2^. The threshold expression of *F*^2^ > σ(*F*^2^) is used only for calculating *R*-factors(gt) *etc*. and is not relevant to the choice of reflections for refinement. *R*-factors based on *F*^2^ are statistically about twice as large as those based on *F*, and *R*- factors based on ALL data will be even larger.

### Fractional atomic coordinates and isotropic or equivalent isotropic displacement parameters (Å^2^)

**Table:**

	*x*	*y*	*z*	*U*_iso_*/*U*_eq_	
Sn1	0.67183 (3)	0.15366 (3)	0.10133 (2)	0.03534 (10)	
O1	0.8345 (3)	0.2746 (3)	0.1623 (2)	0.0437 (7)	
O2	0.6720 (3)	0.3482 (3)	0.2497 (3)	0.0459 (7)	
O3	0.8140 (3)	0.0831 (3)	0.0045 (2)	0.0427 (7)	
O4	0.6338 (3)	−0.0337 (3)	−0.0441 (2)	0.0448 (7)	
C1	0.8867 (4)	0.4597 (4)	0.2822 (3)	0.0333 (9)	
H1	0.9542	0.4175	0.3399	0.040*	
C2	0.8184 (4)	0.5633 (3)	0.3471 (3)	0.0330 (9)	
H2	0.8906	0.6163	0.3888	0.040*	
C3	0.7305 (4)	0.6523 (4)	0.2649 (4)	0.0413 (10)	
H3A	0.6967	0.7202	0.3095	0.050*	
H3B	0.6542	0.6049	0.2246	0.050*	
C4	0.8092 (5)	0.7109 (4)	0.1751 (4)	0.0493 (11)	
H4A	0.7495	0.7637	0.1209	0.059*	
H4B	0.8797	0.7655	0.2149	0.059*	
C5	0.8706 (5)	0.6077 (5)	0.1080 (4)	0.0548 (12)	
H5A	0.7998	0.5575	0.0630	0.066*	
H5B	0.9224	0.6475	0.0539	0.066*	
C6	0.9609 (4)	0.5204 (4)	0.1905 (4)	0.0459 (11)	
H6A	1.0366	0.5694	0.2296	0.055*	
H6B	0.9956	0.4531	0.1457	0.055*	
C7	0.7883 (4)	0.3568 (4)	0.2295 (3)	0.0366 (9)	
C8	0.7475 (4)	−0.0040 (3)	−0.0606 (3)	0.0344 (9)	
C9	0.5448 (4)	0.2734 (4)	−0.0107 (4)	0.0503 (11)	
H9A	0.4773	0.3083	0.0302	0.075*	
H9B	0.5026	0.2249	−0.0768	0.075*	
H9C	0.5962	0.3419	−0.0369	0.075*	
C10	0.6548 (5)	0.0271 (4)	0.2389 (4)	0.0476 (11)	
H10A	0.5894	0.0594	0.2834	0.071*	
H10B	0.7401	0.0197	0.2885	0.071*	
H10C	0.6271	−0.0556	0.2077	0.071*	

### Atomic displacement parameters (Å^2^)

**Table:**

	*U*^11^	*U*^22^	*U*^33^	*U*^12^	*U*^13^	*U*^23^
Sn1	0.03871 (17)	0.03394 (16)	0.03343 (16)	0.00183 (14)	0.00588 (11)	0.00006 (13)
O1	0.0425 (16)	0.0383 (17)	0.0494 (18)	0.0012 (14)	0.0043 (14)	−0.0157 (14)
O2	0.0424 (17)	0.0444 (17)	0.0523 (18)	−0.0088 (14)	0.0116 (14)	−0.0093 (14)
O3	0.0429 (16)	0.0443 (17)	0.0414 (16)	−0.0032 (14)	0.0083 (13)	−0.0124 (13)
O4	0.0463 (17)	0.0475 (17)	0.0436 (17)	−0.0052 (14)	0.0167 (14)	0.0006 (13)
C1	0.031 (2)	0.030 (2)	0.038 (2)	0.0017 (17)	0.0040 (17)	−0.0027 (17)
C2	0.035 (2)	0.027 (2)	0.036 (2)	−0.0024 (16)	0.0042 (17)	0.0000 (16)
C3	0.042 (2)	0.035 (2)	0.048 (2)	0.003 (2)	0.0098 (19)	0.0068 (19)
C4	0.051 (3)	0.044 (3)	0.054 (3)	−0.003 (2)	0.011 (2)	0.016 (2)
C5	0.065 (3)	0.058 (3)	0.044 (3)	−0.011 (2)	0.018 (2)	0.006 (2)
C6	0.042 (2)	0.049 (3)	0.050 (3)	−0.009 (2)	0.018 (2)	−0.009 (2)
C7	0.042 (2)	0.034 (2)	0.032 (2)	0.0055 (19)	0.0026 (18)	0.0016 (18)
C8	0.046 (2)	0.025 (2)	0.031 (2)	0.0025 (18)	0.0038 (18)	0.0057 (16)
C9	0.047 (3)	0.051 (3)	0.052 (3)	0.002 (2)	0.003 (2)	0.011 (2)
C10	0.058 (3)	0.045 (3)	0.041 (2)	0.004 (2)	0.009 (2)	0.007 (2)

### Geometric parameters (Å, °)

**Table:**

Sn1—O3	2.089 (3)	C3—H3A	0.9700
Sn1—C9	2.089 (4)	C3—H3B	0.9700
Sn1—C10	2.098 (4)	C4—C5	1.516 (6)
Sn1—O1	2.102 (3)	C4—H4A	0.9700
Sn1—O4	2.570 (3)	C4—H4B	0.9700
Sn1—O2	2.660 (3)	C5—C6	1.517 (6)
O1—C7	1.294 (4)	C5—H5A	0.9700
O2—C7	1.235 (5)	C5—H5B	0.9700
O3—C8	1.298 (4)	C6—H6A	0.9700
O4—C8	1.232 (5)	C6—H6B	0.9700
C1—C7	1.523 (5)	C8—C2^ii^	1.509 (5)
C1—C6	1.530 (5)	C9—H9A	0.9600
C1—C2	1.540 (5)	C9—H9B	0.9600
C1—H1	0.9800	C9—H9C	0.9600
C2—C8^i^	1.509 (5)	C10—H10A	0.9600
C2—C3	1.514 (5)	C10—H10B	0.9600
C2—H2	0.9800	C10—H10C	0.9600
C3—C4	1.532 (6)		
			
O3—Sn1—C9	106.41 (15)	C5—C4—C3	111.2 (4)
O3—Sn1—C10	109.39 (15)	C5—C4—H4A	109.4
C9—Sn1—C10	137.14 (18)	C3—C4—H4A	109.4
O3—Sn1—O1	80.02 (10)	C5—C4—H4B	109.4
C9—Sn1—O1	102.72 (15)	C3—C4—H4B	109.4
C10—Sn1—O1	105.95 (15)	H4A—C4—H4B	108.0
O3—Sn1—O4	54.83 (10)	C6—C5—C4	110.9 (4)
C9—Sn1—O4	91.89 (15)	C6—C5—H5A	109.5
C10—Sn1—O4	89.93 (14)	C4—C5—H5A	109.5
O1—Sn1—O4	134.84 (9)	C6—C5—H5B	109.5
O3—Sn1—O2	133.28 (9)	C4—C5—H5B	109.5
C9—Sn1—O2	83.34 (15)	H5A—C5—H5B	108.1
C10—Sn1—O2	88.85 (14)	C5—C6—C1	112.1 (3)
O1—Sn1—O2	53.39 (9)	C5—C6—H6A	109.2
O4—Sn1—O2	171.54 (9)	C1—C6—H6A	109.2
C7—O1—Sn1	105.3 (2)	C5—C6—H6B	109.2
C7—O2—Sn1	80.6 (2)	C1—C6—H6B	109.2
C8—O3—Sn1	102.9 (2)	H6A—C6—H6B	107.9
C8—O4—Sn1	82.2 (2)	O2—C7—O1	120.5 (4)
C7—C1—C6	111.9 (3)	O2—C7—C1	123.7 (4)
C7—C1—C2	112.1 (3)	O1—C7—C1	115.8 (3)
C6—C1—C2	110.8 (3)	O4—C8—O3	119.6 (4)
C7—C1—H1	107.3	O4—C8—C2^ii^	124.3 (3)
C6—C1—H1	107.3	O3—C8—C2^ii^	116.1 (3)
C2—C1—H1	107.3	Sn1—C9—H9A	109.5
C8^i^—C2—C3	113.7 (3)	Sn1—C9—H9B	109.5
C8^i^—C2—C1	110.9 (3)	H9A—C9—H9B	109.5
C3—C2—C1	112.7 (3)	Sn1—C9—H9C	109.5
C8^i^—C2—H2	106.3	H9A—C9—H9C	109.5
C3—C2—H2	106.3	H9B—C9—H9C	109.5
C1—C2—H2	106.3	Sn1—C10—H10A	109.5
C2—C3—C4	111.0 (3)	Sn1—C10—H10B	109.5
C2—C3—H3A	109.4	H10A—C10—H10B	109.5
C4—C3—H3A	109.4	Sn1—C10—H10C	109.5
C2—C3—H3B	109.4	H10A—C10—H10C	109.5
C4—C3—H3B	109.4	H10B—C10—H10C	109.5
H3A—C3—H3B	108.0		

Symmetry codes: (i) *x*, −*y*+1/2, *z*+1/2; (ii) *x*, −*y*+1/2, *z*−1/2.
